# Random Illumination Microscopy: faster, thicker, and aberration-insensitive

**DOI:** 10.1038/s41377-024-01687-9

**Published:** 2025-01-02

**Authors:** Boya Jin, Peng Xi

**Affiliations:** 1 Department of Biomedical Engineering, College of Future Technology, Peking University, Beijing, 100871 China; 2 National Biomedical Imaging Center, Peking University, Beijing, 100871 China

**Keywords:** Super-resolution microscopy, Imaging and sensing

## Abstract

The Extended Depth of Field (EDF) approach has been combined with Random Illumination Microscopy (RIM) to realize aberration-insensitive, fast super-resolution imaging with extended depth, which is a promising tool for dynamic imaging in larger and thicker live cells and tissues.

Fluorescence microscopy is leading biological research into a stage where the subcellular dynamic observations in live cells are possible. For exploring the dynamic imaging of larger and thicker three-dimensional (3D) samples, ideal techniques are expected to exhibit outstanding performance in terms of spatial-temporal resolution, 3D field of view (FOV), phototoxicity, and optical sectioning capability, which are extremely challenging to be optimized simultaneously^1^(Fig. 1). Various techniques suitable for live-cell imaging such as laser scanning confocal microscopy (LSCM)^2,3^, two-photon microscopy^4,5^, and light-sheet microscopy^6,7^, have made their efforts to be superior on optical sectioning capability, deeper penetration depth and lower phototoxicity. However, these techniques are based on sequential acquisition along certain dimensions for volumetric imaging, which slows down the imaging speed and makes it difficult to capture dynamic events in live cells. Moreover, the spatial resolution is limited by diffraction, losing the opportunity of observing subcellular organelles. Light-field microscopy^8,9^ as another typical representative of live-cell imaging techniques, can achieve single-frame volumetric imaging with low phototoxicity, but its spatial resolution is lower than wide-field imaging.

**Fig. 1:**
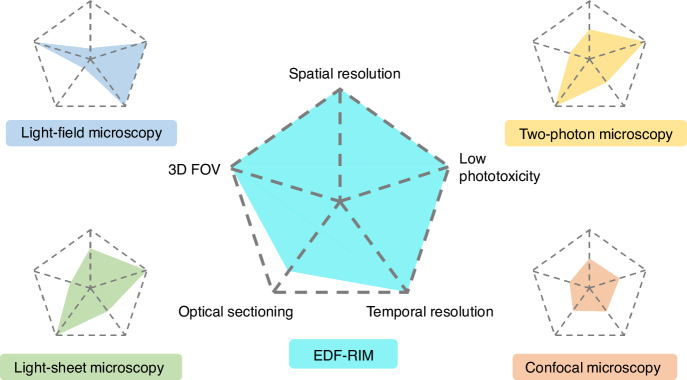
Comparison of live-cell imaging performance in terms of spatial-temporal resolution, low phototoxicity, 3D field-of-view (FOV) and optical sectioning capability between various techniques such as light-field microscopy, confocal microscopy, two-photon microscopy, and light-sheet microscopy. EDF-RIM has demonstrated a comprehensive excellent performance for imaging of larger and thicker live cells or tissues

Super-resolution microscopy, which is able to surpass the limitation of diffraction and distinguish intricate subcellular structures, has attracted the attention of biologists. Structured illumination microscopy (SIM)^10,11^, as the representative super-resolution technique being most suitable for live-cell imaging, can improve the lateral resolution by a factor of two compared to the diffraction limit with lower phototoxicity and faster imaging. Recent research showed that SIM has been successfully utilized in recording subcellular organelles such as mitochondrial cristae^12^, migrasomes^13^, and cytoskeletal interactions^14^, validating its key role in super-resolution live-cell imaging.

However, the super-resolved SIM image is based on computation of multiple images under periodic illumination pattern by translation and rotation, making it highly sensitive to aberrations and suffering from artifacts^15^. Although various attempts have been reported to eliminate artifacts^16,17^, this still significantly limits the uses of SIM in live-cell or tissue imaging. Additionally, SIM requires multiple lateral acquisitions for resolution enhancement and sequential imaging for volumetric recording, which slows down the imaging speed. Addressing such two issues is the key for further advancing the utilization of SIM in live-cell dynamic imaging.

In a newly published paper in Light: Science & Applications, Anne Sentenac’s group from the Aix Marseille University, France, has proposed a new approach to address the two major constraints of SIM^18^. The essence of the idea is the combination of Random Illumination Microscopy (RIM) with Extended Depth of Field (EDF) approach based on optical projection. RIM, as a variant of SIM, replaces the periodic illumination pattern with random speckled illumination, which is able to achieve a two-fold super-resolution enhancement and robust against aberrations^19^. And by compressing the information of samples along the axial direction using optical projection, the EDF during single camera exposure can be realized by rapid scanning of series focal planes with electrically tunable lens (ETL)^20^ or deformable mirror^21^. Such intelligent combination of these two techniques enables SIM to take the lead in the field of dynamic super-resolution imaging of larger and thicker tissues.

Specifically, they achieved a super-resolution reconstruction with a 1.7-fold enhancement over wide-field microscopy by RIM algorithm and the EDF up to 20 μm during single camera integration by using 3D-speckle illumination combined with ETL for rapid scanning of the focal plane. They demonstrated the fast super-resolution imaging capability of EDF-RIM on a 6 μm thick mouse intestinal epithelium, showing a 14-fold increasing of speed and reduced phototoxicity compared to 2D-RIM. They also captured the myosin-based contractile cytokinetic rings in gastrulating Drosophila embryos within 13 μm thick region, confirming that the dynamic contraction of the rings can be smoothly and nicely resolved by EDF-RIM, which would appear bumpy and hard-to-track using 2D-RIM based on sequential acquisition along axial direction. Of course, the EDF technique based on optical projection sacrifices the topography information of the sample. Consequently, they reached a balance between imaging speed and resolution, performing one layer-by-layer scan and extracting topographic data.

EDF-RIM takes advantage of 3D-speckle illumination as its statistical characteristic remains invariant along the optical axis and insensitive to aberrations. Combined with the principle of optical projection, EDF information of sample can be collected within single camera exposure time by rapid scanning of focal plane, enabling fast volumetric imaging and aberration-insensitive super-resolution reconstruction. A prominent advancement of such combination is that the RIM reconstruction algorithm demonstrated a considerable reduction in background from which the classical EDF approaches usually suffer. While the EDF-RIM is based on the assumption that fluorophores are sparsely distributed along the optical axis, making a 3D-speckle illumination suitable for this scenario. Therefore, a possible attempt for the future could be the use of Bessel-speckle illumination^22^, which would allow super-resolution imaging of thicker samples with arbitrary topography, greatly expanding the application prospects of EDF-RIM. Besides improving the volumetric imaging speed, considering RIM is a variant of SIM relying on reconstruction, attention should also be given to enhancing the reconstruction speed, which can be a determining factor for detection and localization of critical events. To achieve this goal, exploring the possibility of reducing the frames of raw images could be beneficial^23^. We anticipate that EDF-RIM, with aberration-insensitive super-resolution imaging for larger and thicker samples, will play a more important role in high spatiotemporal resolution live-cell imaging.
